# Nutrition1Read before the Students’ Association of the McKillip Veterinary College.

**Published:** 1896-07

**Authors:** E. Merillat

**Affiliations:** Vet. Student


### NUTRITION.[1]

By E. MERILLAT, Vet. Student.

NUTRITION, in the light we wish to consider it, is the acme of the physiological process of alimentation, assimilation, and respiration, the true nature of which, whether in highly organized parts, such as the brain, or in tissues, such as cartilage, nail, or horn, is unknown.

These processes are physiological puzzles, which, it is to be feared, will always be beyond the grasp of science. They constitute one of the mysteries always in the mind of every student of physiology, and may be compared with the question of the soul and its relations to the finite and the infinite, which humanity has been either compelled to admit upon the faith of revelation or to hopelessly abandon. It will always be interesting and satisfactory to those acquainted with Nature's laws and willing to accept theories upon the subject concerning which it is impossible in the present condition of science to give any positive information.

It has long been known that certain nitrogen compounds of the organism seem to have the power of reproduction or self-generation ; and it seems that such a compound as protoplasm, with all the wonderful properties ascribed to it, would give us enough information concerning the formation of tissues to enable us to understand the process of nutrition, but, to the contrary, it simply covers up a multitude of unexplained processes between assimilation and disassimilation.

It is a known fact that some tissues undergo decomposition, and that the end-products are excreted, while others, when once

[1] Read before the Students' Association of the McKillip Veterinary College.

formed, undergo no changes, but are gradually desquamated. It is also a well-established fact that the nature of nutritive force or energy resolves itself into vitality, and that the living organism, to a certain extent, depends upon the vitality of the tissues.

Life is always attended with what is known as the phenomena of nutrition, and nutrition does not exist, except in living tissue, and yet there are few if any definitions for "life" which are not open to many grave objections. If we regard life as a principle, it stands as a cause of nutrition; but if we consider it as a result of nutrition, it is an effect. If we study a fecundated ovum in its development, it seems to have a principle which gives it the property of appropriating matter from the outside until it develops from a microscopic to a large body, and this body again develops to produce other ova, and thus perpetuate the existence of its species. It may seem that the organism dies, physiologically, because it has definite form and size, definite period of existence, etc.; but it produces an element capable of perpetuating life in a new being.

The principal forms of life which may interest us under this head are, viz.: First, the life of a fecundated ovum; secondly, the life of tissues; and, lastly, the life of the animal organism.

1. The life of a fecundated ovum is the property which enables it to develop when placed under favorable conditions.

2. The vitality of tissues during development is the property by which it arrives at its perfection to perform its function; and, when perfected, its life is the property which enables it to regenerate itself and perform its function.

3. The life of the animal organism is the sum of all vitalities of its constituent parts. A being may exist without certain parts of the organism without losing its identity. Also an animal may live a long time without consciousness, or an organ paralyzed, or its functions destroyed; but certain functions, such as circulation and respiration, are indispensable to the process of nutrition in all parts; and, as the vitality of the tissue is soon lost when either of these processes are stopped, the animal soon dies. Therefore, we conclude that respiration is a process which assists in nutrition.

We know that tissues have the property of appropriating oxygen and giving off $CO_2$ independently of the blood, and that arterial blood carries oxygen from the lungs to the tissues, and that venous carries $CO_2$ to the lungs to be exhaled. Now the question arises whether the action of oxygen in the economy is the same as in inorganic bodies outside the animal organism. It must be remembered that in the organism we are dealing with compounds that have the property of self-regeneration, and that as a simple condition of normal existence they consume oxygen and give up $CO_2$.

Without the proper supply of oxygen the tissues soon die, lose their properties, and finally disappear by decomposition. The consumption of oxygen cannot be regarded in any other light than as an appropriation of an element necessary to supply waste, and that it is appropriated in the same way as those materials which are nutritive. There can be no doubt that waste is continually going on in the tissues, and as the production of urea, creatin, creatinin, cholesterin, etc., is to a certain extent independent of the absorption of food, so is the production of $CO_2$ in a certain degree independent of the absorption of oxygen.

We can plainly see the difference between the action of oxygen in the economy and that attending the union and decomposition of inorganic matter. For example, by heating $CaCO_3$ we have a new compound ($CaO$), with entirely different appearance and properties, and the liberation of $CO_2$. If we treat any carbonate with an acid we liberate $CO_2$ and have a salt of the acid which has no resemblance to the original compound. Or, if we expose iron to oxygen, it loses its identity, and the compound formed is the oxide of iron, or iron-rust. A still more striking change takes place in the combustion of fats, the products of which are $CO_2$ and water, and the fat disappears. In the living organism the organic compounds are continually changing, breaking down, and forming various excrementitious principles, at the head of which is $CO_2$, but the tissue always remains the same, although to ignore the combustion theory altogether would simply be denying a fact. It is essential that the tissues maintain a certain chemical integrity, which requires a supply of new material as food, and, above all, a supply of oxygen; therefore it may not be wrong to call oxygen a nutritive element (?).

It is sometimes said that respiration is a slow process of combustion, but when we examine the blood we learn that oxygen is not consumed in the lungs, but is carried to the tissues by the circulation, and that $CO_2$ is brought to the lungs to be exhaled. These facts, in connection with the fact that the tissues consume oxygen and give off $CO_2$, lead us to change the location of combustion from the lungs to the tissues. We know that the organic principles of the body which form the bases of tissues are continually changing in their normal condition; that after a period of existence they degenerate into excrementitious matter, and are regenerated by a change of material furnished by the blood. As far as respiration of these parts, we can only say that oxygen is consumed and $CO_2$ is liberated. The process by which this is done is as complex as that of nutrition, and so far no one has been able to give a reason for the absorption of oxygen and the exhaling of $CO_2$, or to explain the condition of oxygen or what is lost in the formation of $CO_2$.

The questions which naturally arise are: 1. How is oxygen consumed? 2. How is $CO_2$ produced? 3. What takes place between the consumption of oxygen and the evolution of $CO_2$?

Oxygen is supposed to be absorbed by the red blood-corpuscles and to enter into their composition and be carried to the tissues, where it is consumed with the nutritive material in the circulation. We know very little of the laws which regulate the consumption of oxygen, and are not able to find out the exact changes resulting from the action of it upon the tissues. There is no evidence in favor of the view that oxygen unites directly with the carbon in the blood which it meets in the lungs to form $CO_2$; but it is more plausible that it unites with the organic principles of the system and supplies the imperative want, which is felt by all animals and in all parts of the body. The fact that $CO_2$ can be produced artificially does not prove that its formation in the body is as simple as when formed by combustion. We are not justified in supposing that the formation of $CO_2$ in the process of nutrition is the same as when produced by chemical means. It is more reasonable to suppose that it is produced as a product of excretion. The $CO_2$ thus formed is taken up by the blood; part of it in a free state and part in solution is given off by the lungs in simple displacement, and the remainder combines with the carbonates to form bicarbonates. We conclude, therefore, that $CO_2$ orginates in the tissues as a product to be excreted in the process of nutrition, but we do not know what effect its evolution has upon them.

We also know that oxygen is consumed by the tissues as an element to supply waste, but its immediate effects are unknown. When we can tell what occurs in the tissues between the assimilation of oxygen and the evolution of $CO_2$ our knowledge of respiration and nutrition will be complete, but this at present no one is able to explain. We only know that oxygen is a principle of nutrition and that $CO_2$ is a product of excretion. The immediate process between the two belongs to the function of nutrition, and we know nothing of its immediate nature. We have not enough evidence to suppose that the process is the same as that known as combustion.

We can scarcely realize the extent of the problem of nutrition from a review of the functions we have already considered. We know that blood contains all the elements that enter into the composition of tissues, either identical as organic principles or in a form that will allow them to be changed into principles of the tissues as organic principles, and that these principles are supplied to the tissues by circulation, and are continually drawn through the tissues to supply them with raw material for their regeneration, and are kept to the proper standard by the introduction of new matter into the system by alimentation, etc. The introduction of the new matter is in demand on account of the changes of the substance of the tissues into what we commonly call waste or worn-out matter, and this is discharged from the animal organism to be appropriated by the vegetables, and thus maintain the equilibrium between the two great kingdoms.

The inorganic compounds that are taken in as food are discharged in the form that they enter, either as feces, urine, or perspiration, but it must not be inferred from this that they are not useful as constituents of the body. Some of them are more abundant in the fluids than in the solids (NaCl), while others are more abundant in the solids than in the fluids (lime salts), and their function is mechanical.

The organic principles are divided into nitrogenous and non-nitrogenous. The nitrogenized principles contain carbon, hydrogen, nitrogen, and oxygen, sometimes sulphur. These principles are taken into the body and, after undergoing a change in digestion and absorption, are excreted as excrementitious matter. The nature of these series of transformation is unknown, but we know that the deposition of tissues constitutes one important act of nutrition. The modification of nutrition, due to its supply, has a well-defined limit, as, for example, an excess taken as food is not discharged in the feces, nor does it pass off in the form in which it entered, but apparently becomes absorbed by the blood and increases the quantity of urea; whether the quantity not needed in nutrition is transformed into urea in the blood or increases the activity of dissimilation in the tissues is hard to determine; however the excess of inorganic matter is thrown off nearly in the same way as an excess of organic, the difference is that one is excreted as it enters and the other as urea.

The non-nitrogenous elements taken up by the blood are sugars and fats. Sugars consist of carbon, hydrogen, and oxygen in the proportion of water ($H_2O$). It is never discharged from the body in health, and is never deposited in any part of the organism, even as a temporary condition. It can be converted into $CO_2$ and water or lactic acid ($C_6H_{12}O_6 = 2C_3H_6O_3$). It does not enter into the composition of tissues, but may take the place of albuminoids in oxidation to form fats, and is no doubt an important factor in the production of heat ($C_2H_{12}O_2 + 12O = 6CO_2 + 6H_2O$), which may be the source of energy, *i. e.*, part of the heat produced being converted into mechanical force.

The next class of non-nitrogenized principles is fat. It is found in the organism in the form of adipose tissue, and is consumed in the production of heat. It is a principle always found in the body and never discharged as fat. It is formed in the economy by the oxidation of albuminoids and carbohydrates, and may also be taken into the body by absorption. When once in the circulation it is soon destroyed or deposited in the form of adipose tissue. It takes no part in the formation of other tissues, and, in the absence of carbohydrates in the circulation, serves to maintain the temperature of the organism and to generate energy. Therefore we may consider fats and carbohydrates potential energy, and the oxidation of them forming kinetic energy. They occupy an important position between nitrogenized and non-nitrogenized principles, *e. g.*, albuminoids may be oxidized and become fats, and carbohydrates may in the same way become fats, but fats cannot become albuminoids nor carbohydrates; and, again, we also know that in the combustion of fats and carbohydrates the end-products are identical ($CO_2$ and $HO_2$). Therefore it would not be wrong to suppose that their ultimate functions are identical.

We shall now briefly recapitulate and draw a conclusion, and for convenience shall divide the propositions into primary and secondary. The primary propositions are the functions of principles, etc., and are as follows:

1. That the inorganic principles have a function that is purely mechanical.

2. That the function of nitrogenized principles is to form tissues.

3. That the function of non-nitrogenized principles is to produce heat and energy.

4. That the life and regeneration of tissue-cells resemble the life of a fecundated ovum.

The secondary propositions are, viz.:

1. That tissues are composed of cells.

2 That tissues maintain their normal integrity by regeneration of cells.

3. That regeneration of cells resembles the development of a fecundated ovum; and

4. That the cells act upon the nitrogenized principles as the germ or nucleus of an ovum does upon its albumin.

Now the conclusions we can draw from the foregoing propositions are as follows: That we concentrate the processes of nutrition to the proliferation of cells; and that the way in which this is done is attributed to the changes that occur in the protoplasm of the cell. Therefore we see what a useful, wonderful, and indispensable chemical compound protoplasm is to the student of physiology and pathology. In the demonstration of unexplained processes of physiology, and in the explanation of pathological processes or deviation from any normal physiological condition, the scientist always takes refuge in the dark, unfathomed recesses of protoplasm.

